# Medical Appointments

**Published:** 1851-01

**Authors:** 


*Medical Appointments.*—Dr. S. G. Moses, has been elected Prof. of Obstetrics in the Medical Department of the University of Missouri.

Prof. Dugas, who has hitherto occupied the Chair of Physiology and Pathology in the Medical College of Georgia, has been transferred to the Chair of Surgery, in the same institution, made vacant by the resignation of Prof. Eve; and Dr. H. V. M. Miller, of Georgia, has been elected to the Chair vacated by the transfer of Prof. D.

